# IL-20 bone diseases involvement and therapeutic target potential

**DOI:** 10.1186/s12929-018-0439-z

**Published:** 2018-04-24

**Authors:** Hsiao-Hsuan Wang, Yu-Hsiang Hsu, Ming-Shi Chang

**Affiliations:** 1 Institute of Basic Medical Sciences, National Cheng Kung University, Tainan, Taiwan; 2 Institute of Clinical Medicine, National Cheng Kung University, Tainan, Taiwan; 3 Department of Biochemistry and Molecular Biology, College of Medicine, National Cheng Kung University, Tainan, 704 Taiwan; 4 Research Center of Clinical Medicine, National Cheng Kung University Hospital, Tainan, Taiwan

**Keywords:** IL-20, Bone homeostasis, Rheumatoid arthritis, Osteoporosis, Cancer-induced osteolysis, Bone fracture

## Abstract

**Background:**

Millions of people around the world suffer from bone disorders, likes osteoporosis, rheumatoid arthritis (RA), and cancer-induced osteolysis. In general, the bone remodeling balance is determined by osteoclasts and osteoblasts, respectively responsible for bone resorption and bone formation. Excessive inflammation disturbs the activities of these two kinds of cells, typically resulting in the bone loss.

**Main body:**

IL-20 is emerging as a potent angiogenic, chemotactic, and proinflammatory cytokine related to several chronic inflammatory disorders likes psoriasis, atherosclerosis, cancer, liver fibrosis, and RA. IL-20 has an important role in the regulation of osteoclastogenesis and osteoblastogenesis and is upregulated in several bone-related diseases. The anti-IL-20 monoclonal antibody treatment has a therapeutic potential in several experimental disease models including ovariectomy-induced osteoporosis, cancer-induced osteolysis, and bone fracture.

**Conclusion:**

This review article provides an overview describing the IL-20’s biological functions in the common bone disorders and thus providing a novel therapeutic strategy in the future.

## Background

Cytokines participate in numerous physiological events including the regulation of immune and inflammatory responses and embryonic development. They are produced locally and transiently controlling the response duration [1]. In general, cytokines act on the cells in three main patterns, including autocrine (act on cells which secret them), paracrine (act on adjacent cells) and endocrine (function on distant cells) [2]. The dysregulation of cytokine signaling is usually observed in patients with immune-mediated diseases [3]. After produced by active macrophages, proinflammatory cytokines function as upregulating many inflammatory processes. It has been reported that many proinflammatory cytokines are related to the pathogenesis of inflammatory disorders, especially for TNF-α, IL-1β, and IL-6 [4]. Therapeutic strategy regulating the function of these proinflammatory cytokines likes TNF-α, IL-1β, and IL-6, by neutralizing them using blocking antibodies or soluble receptors, have been extensively used over the past decades [5, 6].

Bone homeostasis is retained by the two kinds of bone cells through the bone resorption and bone formation processes [7]. Osteoblast precursor cells come from mesenchymal stem cells (MSCs), and require Runx2 transcription factor for development to the mature osteoblast lineage [8, 9]. Osteoblasts are not only involved in bone formation, they still regulate the differentiation of osteoclast through secreting macrophage colony-stimulating factor (M-CSF), receptor activator of nuclear factor kappa-B ligand (RANKL) and osteoprotegerin (OPG). M-CSF facilitates the development of osteoclast precursor cells and promotes their survival. RANKL is an indispensable factor for osteoclastogenesis which promotes osteoclast differentiation through interacting with the RANK receptor on the precursor cells. In order to against the excessive bone resorption, osteoblasts have the ability to secret OPG, as a soluble decoy receptor, bind to RANKL to suppress the differentiation of osteoclasts [8, 10–12]. Dysregulation of these two types of cells causes bone disorders, likes rheumatoid arthritis (RA) and osteoporosis [7, 13]. Proinflammatory cytokines, which are secreted mainly by macrophages, synovial fibroblasts, and lymphocytes within the inflamed synovium, such as TNF-α, IL-1, IL-6, and IL-17, break the balance of bone homeostasis, typically resulting in a bone loss and erosion [11, 14].

Interleukin-20 (IL-20), a component of IL-10 family, activates the signal transduction via two kinds of heterodimeric receptor complexes, IL-20R1/IL-20R2 or IL-22R1/IL-20R2 [15]. Previous studies indicated that IL-20 has a close connection with psoriasis [16], atherosclerosis [17], and stroke [18]. Recently, it is revealed that IL-20 is a key factor in regulating angiogenesis [19], chemotaxis [20], osteoblastogenesis [21], and osteoclastogenesis [22]. This review mainly focuses on discussing the pathological role of IL-20 in the pathogenesis of bone diseases and the therapeutic potential of anti-IL-20 monoclonal antibody (mAb) in several inflammation-mediated bone loss diseases such as RA, osteoporosis, cancer-induced osteolysis, and bone fracture.

### The discovery of IL-20

IL-20 was first discovered from human keratinocyte library using EST database and structural-based algorithm in 2001 [15]. As a component in the IL-10 family, IL-20 shares structure homology with other members (IL-10, IL-19, IL-20, IL-24, and IL-26). Human and mouse IL-20 have 76% amino acid sequence identity in total 176 amino acids. The human *IL-20* gene maps to a region on chromosome 1 and is close to IL-10, IL-19, and IL-24. There are two nuclear factor κB (NF-κB)-binding sites in the IL-20’s promoter, representing that NF-κB has the ability to drive the transcription of IL-20 [23]. The 3′-untranslated region (UTR) of IL-20 contains mRNA instability motifs, which indicated that IL-20 mRNA has a quick turnover rate.

### Expression pattern of IL-20 and its receptors

IL-20 transcripts are detected in skin, lung, reproductive glands, and kidney of the human body. Tissue microarray analysis shows that IL-20 is preferentially expressed in macrophages, epithelial cells, endothelial cells, myoepithelial cells, and skeletal muscle cells on normal physiological condition [24]. We recently found that IL-20 is upregulated in rheumatoid arthritis synovial fibroblasts (RASFs) [25] and several cancer cells including breast cancer, oral cancer, and prostate cancer [26, 27].

IL-20 binds to two kinds of receptor complexes, including IL-20R1/IL-20R2 and IL-22R1/IL-20R2. Both the heterodimer receptor complexes signals partly through the JAK-STAT pathways [28]. The mRNA expression analysis showed that the expression of IL-20R1, IL-20R2, and IL-22R1 have a limited tissue expression pattern [15].

### Biological functions of IL-20

Both IL-20 and its receptors are expressed in keratinocytes and they are found overexpressed in the human psoriatic skin [15, 29]. IL-20 is able to stimulate the expression of several cytokines involving in inflammatory reactions, for instance, TNF-α and MCP-1 [30]. Therefore, IL-20 seems to be involved in the pathogenesis of psoriasis. IL-20 also promotes IL-1β expression in hypoxic kidney tubule cells [31]. The gene expression of TNF-α, IL-6, and IL-1β are found to be regulated by IL-20 in various cell types, indicating IL-20 is an upstream key regulator in the processes of inflammatory responses [26, 32, 33].

Chemokines function as regulating inflammation, leukocyte trafficking, and the differentiation of immune cells. Chemokines are also important in selectively recruiting monocytes, neutrophils, and lymphocytes [34]. MCP-1 is a typical chemoattractant of T cells and monocytes. IL-8 is also a chemokine able to recruit neutrophils. Our previous studies showed that IL-20 upregulates MCP-1 and IL-8 expression in several types of cells, such as primarily cultured human disc cells and RASFs [25, 33]. IL-20 also induces the neutrophil chemotaxis in vitro [25], indicating IL-20 has chemoattractant properties [35].

Previous studies indicated that IL-20 induces angiogenesis both in a direct and indirect pattern [19]. IL-20 induces the migration, proliferation, and vascular tube formation of human umbilical vein endothelial cells (HUVECs) through regulating matrix metalloproteinase (MMP)-9, vascular endothelial growth factor (VEGF), fibroblast growth factor 2 (FGF2), MMP-2, and IL-8 [19, 36]. In addition, IL-20 promotes tumor angiogenesis in several mouse models. In apolipoprotein E deficient (apoE^−/−^) mice, IL-20 is able to enhance IL-6 and TNF-α expression and accelerate the development of atherosclerosis [17]. Therefore, IL-20 functions as a pleiotropic proinflammatory cytokine, which enhances the inflammation, chemotaxis, and angiogenesis.

### Signal transduction of IL-20

According to the IL-20 and its receptors expression profiles, the signaling activities of IL-20 were revealed. The IL-20 expression is induced by IL-1β via p38 MAPK and NF-κB-dependent pathway [23]. We previously found the hypoxia induces IL-20 expression in human embryonic kidney cells, monocytes, keratinocyte, chondrocytes, and glioblastoma cells [18]. Several studies have reported that IL-20 triggers JAK-STAT3 pathways in HaCaT cells [18] and renal baby hamster kidney fibroblasts transfected with IL-20 receptors, IL-20R1 and IL-20R2 [15]. IL-20 induces the translocation of STAT3 from cytosol to nuclear in HaCaT cells [15]. IL-20 also activates different signal pathways in different cell types, such as extracellular signal-regulated kinase 1/2 (ERK1/2) in RASFs [25] and p38, JNK, ERK1/2 in HUVECs [19]. IL-20 was shown to promote the activation of JNK, ERK and STAT3 and induced apoptosis through caspase 9 activation in HK-2 (human proximal tubular epithelial cells) [31]. IL-20 induces the expression of MMP-9 and activates ERK1/2, p38 MAPK, JNK, and JAK-STAT signaling pathways in bladder cancer cells [37].

## IL-20 and bone diseases

### IL-20 in rheumatoid arthritis

As a systemic autoimmune disease, RA affects approximately 1% population worldwide. In RA, the immune system mistakenly attacks joints and causes inflammation, resulting in swelling and pain and bone destruction [38]. Although the cause of RA is controversial and heterogeneous, it’s believed that the major mechanism of RA is chronic inflammation [39]. One of the main characteristics of RA is the proliferating synovial fibroblasts and infiltrating immune cells in synovial joints. Several studies revealed the RASFs play an important role in RA initiation through secreting matrix-degrading enzymes (MMPs), chemokines, and cytokines, likes TNF-α, IL-6, IL-1β, and granulocyte-macrophage-colony-stimulating factor (GM-CSF) [40].

Angiogenesis is also a hallmark in the early stage of RA pathogenesis, which enhancing immune cells into the synovial tissue [41]. It is observed that several angiogenic factors such as VEGF and FGF2 are highly expressed in RASFs [42]. In our previous study, IL-20 is also an angiogenic factor which induces the migration, proliferation, and vascularization both in vitro and in vivo [19]. Based on these observations, we hypothesize that IL-20 may be an upstream mediator in the RA progression.

We and other groups reported that the level of IL-20 in synovial fluid was significantly higher in RA patients than in OA patients,, while no difference was observed in serum levels of IL-20 between these groups [25, 43, 44]. However, one study revealing no difference was observed on the IL-20 level in synovial fluid between RA and OA patients [45]. The discrepancies might be due to some differences in the disease stages, drug-use history, and experimental procedure.

In our previous study, both IL-20 and its receptors are detected in RA synovial membranes and fibroblast-like synoviocytes (RA FLS) isolated from RA patients. IL-20 promotes the growth of endothelial cells (HUVECs), the migration of RA FLS and the chemotaxis of immune cells [25]. Furthermore, IL-20 stimulates the expression of IL-6, IL-8 and MCP-1 in RA FLS through ERK-1/2 signaling activation [25]. Other group showed that proinflammatory cytokines, including TNF-α and IL-1β, induced IL-20 expression in RA FLS [46]. These results indicated IL-20 is a critical mediator in the proinflammatory cytokine cascade and contributes to joint inflammation in the pathogenesis of RA. A recent study reported that the secretion of IL-20 in RA may be stimulated by TLR ligands. The level of IL-20 was induced in RA patients’ PBMCs by TLR ligands, such as Poly I:C and LPS, rather than the proinflammatory cytokines, such as TNFα and IL-1β [43]. Therefore, the secretion of IL-20 may be independent of the proinflammatory cytokines in RA. IL-20 is not only a chemoattractant by itself but also an inducer of other chemokines. The data indicate that IL-20 is a determinant factor in RA and IL-20 antagonists may have potential therapeutic effects for RA [35].

We generated collagen-induced arthritis (CIA) experimental model to examine the role of IL-20 in RA. Based on the observation that both IL-20 and IL-20R1 are highly expressed in the CIA rats’ synovial tissue, we intra-muscularly electroporated soluble IL-20R1 (sIL-20R1) plasmid DNA to block IL-20’s function through inhibiting IL-20R1 signaling and found that the arthritis severity of the CIA rats was significantly reduced. Anti-IL-20 monoclonal antibody (mAb) 7E significantly improves the symptoms of arthritis through reducing thickness and swelling of hind-paw, suppressing bone destruction, and inhibiting the production of proinflammatory cytokines in synovial tissue.

It is known that TNF-α, IL-20, IL-6, and IL-1β are all involved in the pathogenesis of RA, and RANKL is also regarded as a key factor in bone erosion in RA [47]. MMPs, matrix-degrading enzymes secreted by RASFs (Rheumatoid arthritis synovial fibroblasts), are considered a key factor in RA initiation [48]. The anti-IL-20 mAb 7E downregulates the proinflammatory cytokines (IL-20, IL-6, and IL-1β), RANKL and matrix metalloproteinase (MMP-1, − 3, and − 13) in the CIA rats’ synovial tissues [49]. This result demonstrates anti-IL-20 mAb 7E both reduce the symptoms of arthritis and help to prevent bone destruction.

In RA, osteoclastic bone resorption typically causes bone erosions at the sites of synovitis that rich in RANKL. RA FLS and osteoblasts are the cellular sources of RANKL production. A recent study reported that IL-17 produced by Th17 cells acts on osteoblasts to promote bone destruction [50]. IL-20 enhances the expression of RANKL and IL-17 in osteoblastic MC3T3-E1 cells, suggesting IL-20 might be a critical factor in the osteoclastogenesis through regulating RANKL/RANK axis [51]. According to the data, we proposed IL-20 is closely related to the progression of RA. Therefore, targeting IL-20 may significantly improve the severity of arthritis.

### IL-20 in osteoporosis

Osteoporosis is a critical risk factor for fragility fractures, causing substantial morbidity and mortality, especially for elders [8]. Osteoporosis occurs when the speed of osteoclastic bone resorption goes beyond that of the osteoblastic bone formation, and typically accompanied by the consequences of the loss of bone mineral density (BMD) and a decrease of bone structure and strength.

We previously observed that concentration of IL-20 in serum was obviously higher in osteopenia and osteoporosis patients compared to healthy ones, suggesting IL-20 might involve in the bone loss [22]. In ovariectomy (OVX)-induced osteoporotic mice, anti-IL-20 mAb 7E treatment not only increased the tibia BMD but also decrease the number of osteoclasts on the bone surface. Moreover, the C-terminal telopeptide of collagen (CTX), which is the bone resorption marker, was found downregulated in the serum after anti-IL-20 mAb 7E treatment.

Excessive osteoclast activity leads to bone loss in osteoporosis. The osteoclast differentiation needs the upregulation of M-CSF and RANKL [52, 53]. Both M-CSF and RANKL are believed to be essential and sufficient for the differentiation of osteoclast. A previous study [54] indicated that IL-20 was not involved in differentiation of osteoclasts from synovial fluid cells. However, they incubated IL-20 alone in synovial fluid cells without M-CSF and RANKL. Thus, their culture system may not be suitable for osteoclast differentiation. In our in vitro osteoclast differentiation system, we found that IL-20 alone without M-CSF and RANKL did not trigger osteoclast differentiation. However, we also found that IL-20 is critical for osteoclast differentiation; our evidence is that anti-IL-20 mAb 7E completely inhibited osteoclast differentiation in the presence of M-CSF and RANKL. Therefore, although M-CSF and RANKL are considered to be enough for osteoclastogenesis in general, our data demonstrated that IL-20 is another critical factor involving in osteoclast differentiation.

Both osteoclasts precursor cells and mature osteoclasts express IL-20 and IL-20’s receptor, showing IL-20 may act on the autocrine manner in these cells. The interaction between RANKL and RANK is essential to the differentiation of osteoclast [12]. When RANKL binding to the RANK receptor on osteoclast precursor cells, the activated signal drives osteoclastogenesis from hematopoietic progenitor cells to mature osteoclasts [10, 12]. IL-20 has the ability to upregulate the expression of RANK and several markers of osteoclast differentiation (likes NFATc1, c-Fos, cathepsin K, and TRAP) in osteoclast precursor cells. While these activities were inhibited after anti-IL-20 mAb 7E treatment. Previous study showed that cathepsin G protease which is produced by osteoclasts, cleaves the extracellular domain of RANKL from osteoblasts and releases soluble RANKL (sRANKL) to promote osteoclastogenesis [55]. We found that IL-20 induces cathepsin G secretion in osteoclasts and modulated sRANKL formation. Furthermore, IL-20 also induces the RANKL expression in preosteoblasts. The observations support that IL-20 modulates osteoclast differentiation through multiple different pathways.

In the OVX-induced osteoporotic model, blocking IL-20’s function by treating anti-IL-20 mAb 7E or by using IL-20R1 deficiency showed a protective effect against bone destruction. Therefore, the strategy of blocking IL-20 may help us fight against osteoporosis.

### IL-20 in cancer-induced osteolysis

As a leading cause of cancer death among women, breast cancer remains a serve public health issue worldwide [56]. Nowadays, the therapeutic strategies targeting primary breast tumor are improved obviously, but treatments for metastasis are still a big issue [57]. Metastatic breast cancer accounts for the majority of deaths from breast cancer [58]. It was reported that about 85% of advanced stage breast cancer patients have bone metastasis, resulting in incurable osteolysis [59].

We previously found that both IL-20 and its receptors (IL-20R1 and IL-20R2) are overexpressed in the sites of primary tumor and bone-metastasis in breast cancer patients, and the higher expression of IL-20 is correlated with poorer clinical outcome [27].

There are many cytokines, MMPs and proteases secreted by tumor microenvironment to help cancer metastasize to distant organs [60, 61]. Previous studies [37, 62, 63] reported that MMP-12, MMP-9, and cathepsin K accelerate extracellular matrix (ECM) degradation leading the invasion and metastasis of cancer cells. We found that IL-20 is able to stimulate the growth, migration, and function as an upstream mediator to enhance MMP-9, MMP-12, cathepsin K, and cathepsin G secretion in cancer cells. These proteases are implicated in the interaction between tumor and stroma and help cancer to metastasize. Therefore, IL-20 might provide a suitable microenvironment for breast cancer metastasis.

The crosstalk between tumor cells, osteoblasts, osteoclasts, and bone extracellular matrix form a vicious cycle for the initiation and development of metastatic lesions in the skeleton. In breast cancer-induced osteolytic bone metastasis model, anti-IL-20 mAb 7E treatment not only inhibited the bone colonization but also protested against the osteolytic bone loss in mice. The data suggest that IL-20 is a key factor involved in the progression of breast cancer and anti-IL-20 mAb has a therapeutic potential to protect breast-cancer induced osteolysis.

Prostate cancer is the third leading cause of death among men worldwide. The prostate cancer patients died mainly because of the bone metastasis and frequently accompanied by the serve symptoms of bone pain and bone fracture [64, 65]. Epithelial-mesenchymal transition (EMT) is a cellular event describing epithelial cells losing connection with each other and dedifferentiate to acquire mesenchymal phenotype, which enhances the migratory and invasive abilities [66–68]. After the EMT, cancer cells begin to leave the primary site into the bloodstream, entry into the vascular system and undergo extravasation from the circulation to distant sites [69].

Cancer cells secrete IL-1, IL-6, and M-CSF to stimulate the activation of osteoclast [70, 71]. These cytokines also act on osteoblast to induce RANKL expression. After binding with RANK on osteoclasts, RANKL through signaling activation to induce the maturation of osteoclasts [10, 72]. The active osteoclasts and the prostate cancer cells secrete the proteolytic enzyme cathepsin K to degrade the ECM and contribute to bone resorption and metastasis [73]. Both cathepsin G and cathepsin K play crucial roles in tumor-induced osteolysis, and they are also potentially a novel therapeutic target for treating cancer [74, 75].

IL-20 and its three receptors transcripts are detected in the human prostate adenocarcinoma tissue samples, indicating that prostate cancer is one of the targets of IL-20. In vitro, IL-20 significantly enhances the proliferation, migration, and anchor-independent growth in prostate cancer cells [76]. IL-20 is involved in the EMT through ERK1/2, AKT, p38, and NF-κB pathways by inhibiting epithelial marker E-cadherin expression, and promoting mesenchymal marker N-cadherin, STAT3, vimentin, and fibronectin expression. We also found that IL-20 enhances RANKL production through regulating cathepsin G and cathepsin K in prostate cancer cells [76]. In vivo, anti-IL-20 mAb 7E not only shrank the tumor mass but also prevented prostate cancer-induced bone loss [76]. The data demonstrate that IL-20 masters in regulating of cancer-induced bone osteolysis.

### IL-20 in bone fracture

Bone fracture is the most frequent traumatic injuries in human [77]. The fracture healing process can be divided into three determinant stages, inflammation, repair and remodeling [78].

Fracture healing is a complex and a dynamic process being governed by a variety of cellular elements and cytokines, in which osteoclasts and osteoblasts are the two important cell types. Osteoclasts are responsible for cartilage resorption and remodeling during fracture healing by secreting acid and proteinases. On the other side, osteoblasts synthesize the bone matrix and promote mineralization [8, 9, 79]. IL-20 and its receptors transcripts were detected in osteoclasts, osteoblasts, and chondrocytes in the bone fracture callus [21]. Also, we found the IL-20 is upregulated in the serum of bone fracture animal model, and anti-IL-20 mAb 7E treatment enhanced bone formation. Anti-IL-20 mAb 7E treatment not only raised the number of osteoblasts but also inhibited the number of osteoclasts at the bone callus during fracture healing. The data indicate that IL-20 has the ability to inhibit osteoblastogenesis and promotes osteoclastogenesis. IL-20 is a determinant factor in regulating the balance between the differentiation of osteoclast and osteoblast. Bone homeostasis is a combination of osteoclast and osteoblast activity. We found IL-20 has an inhibitory effect on the osteoblast maturation from mouse preosteoblastic MC3T3-E1 cells [21]. However, others have found IL-20 induced the mineralization of primary human osteoblasts [80]. The effect of these different mechanisms needs further clarification.

We used IL-20R1 deficiency mice to study the importance of IL-20R1 during bone fracture healing. The fractures healing rate of IL-20R1^−/−^ mice showed more quickly than wild-type. Furthermore, the osteoclast number in the bone surface is less in the IL-20R1^−/−^ mice than wild-type mice. The results suggest that IL-20/IL-20R1 signaling mediates bone homeostasis and is involved in metabolic bone disease.

Sclerostin is reported to inhibit bone formation through downregulating the differentiation, the proliferation and the function of osteoblast function [81, 82]. IL-20 and sclerostin are positively correlated in the serum of bone fracture, osteopenia, and osteoporosis patients. Furthermore, IL-20 targeted osteoprogenitor MC3T3-E1 cells to enhance sclerostin expression and inhibit OPG expression. By using human amniotic fluid-derived cells to study the role of IL-20 in osteoblastogenesis, IL-20 could inhibit the gene expression of multiple osteoblast differentiation factors, such as osterix (OSX), RUNX2, and Atf4. IL-20 not only regulated Wnt/β-catenin signaling to inhibit OSX expression but also directly suppressed OSX promoter activity. The data demonstrate that IL-20 regulates both the pro- and anti- osteogenic processes to inhibit osteoblast maturation [21].

## The therapeutic potential of anti-IL-20 mAb

### Rheumatoid arthritis

As the progress of medical advances, the treatment of RA has changed a lot, from the traditional treatments such as glucocorticoids and nonsteroidal anti-inflammatory drugs (NSAIDs) to the cytokines-based therapy [83]. Since the 1990s, anti-TNF-α mAb has become the major biological drugs for treating RA [84]. However, it is estimated approximately 40% RA patients are not sensitive to TNF blockade therapeutics [85]. Severe arthritis could even be observed in the TNF knockout mice. This evidence indicates some unknown factors involved in the TNF-independent pathway may have critical effects on the RA pathogenesis. Thus, targeting single pathogenic factor is not enough for treating RA [86].

Based on our previous findings, IL-20 modulates several pathways in RA progression: (I) IL-20 induces MCP-1, IL-6, TNF-α, and IL-8 expression in RA FLS, causing the recruitment of neutrophils and promote inflammation [25]. (II) IL-20 functions as an angiogenic factor, IL-20 induces VEGF, FGF2, and MMPs expression in endothelial cells to facilitate the infiltration of immune cells into inflamed synovial tissues [19, 25]. (III) IL-20 functions as an osteoclastogenic cytokine to promote osteoclastogenesis through regulating RANKL, RANK, and IL-17 and then causes bone destruction [49].

We used the CIA rat model to investigate whether the blockade of IL-20 improves the disease activity. The anti-IL-20 mAb 7E treatment significantly ameliorated the arthritis symptoms and prevented CIA rats from bone destruction. Furthermore, the combination of anti-IL-20 mAb 7E with Etanercept (the inhibitor of TNF-α) treatment showed the synergistic effect and was more efficient than the Etanercept treatment alone. Therefore, the combination of anti-IL-20 mAb with other cytokine blockades may also be a promising anti-rheumatic strategy.

In our CIA rat model, both IL-20 and IL-20R1 were upregulated in the synovial tissue compared with healthy control rats [25]. However, one study indicated that IL-20 mRNA expression from the paws and lymph nodes of CIA mice was very low [87]. The mRNA transcripts of IL-20R1 was also low and indicated a negative correlation with paw disease scores [87]. Therefore, very low local expression of IL-20 in the diseased paws and lack of correlation with disease severity may explain the lack of efficacy of anti-IL-20 treatment in the mouse CIA model.

Both rheumatoid factors (RFs) and anti-cyclic citrullinated peptide (anti-CCP) are autoantibodies typically found in the blood and synovial fluid of RA patients, which are used for the early diagnosis of RA [88]. Seropositive (RF- and anti-CCP- positive) RA patients are accounted for about 60–80% RA patients [89, 90]. Recently, the levels of IL-20 were found significantly elevated in the seropositive RA patients than in seronegative RA patients [43, 54]. In addition, the serum and the synovial fluid levels of IL-20 were correlated with the RA disease activity, such as the DAS28 score, the number of swollen and tender joints [43]. In a phase 2a trial (NNC0109–0012), anti-IL-20 mAb seems to be effective particularly in the patients with seropositive RA (RF- and anti-CCP- positive) rather than seronegative RA patients, but the phase 2b trial (NCT01636843) failed because the primary efficacy endpoint was not met [91]. It is not clear if seronegative RA patients were excluded in the phase 2b trial. It’s believed that there are different inflammatory pathways involved in the seropositive and seronegative patients [92]. Based on our previous data, not all the RA patients highly expressed IL-20 in the synovial fluid [25]. The IL-20 level was significantly elevated in the seropositive RA patients compared with that in the seronegative RA patients [43, 54]. Thus, this observation suggests IL-20 is closely related to the autoantibodies (rheumatoid factors and anti-CCP) and may be a key mediator for regulating these factors, which awaits further exploration.

On the other hand, failure in the clinical trial may be due to the redundancy effect in the IL-20 cytokine subfamily [93]. As a member of IL-20 subfamily, IL-19 was also overexpressed in CIA rat model, and treatment of anti-IL-19 antibody significantly ameliorated the severity of arthritis [94]. IL-24, which shares the same receptor complex with IL-20, was also upregulated in synovial fluid and plasma of RA patients [45, 95]. A recent study showed that a broad blockade of IL-20 subfamily (including IL-19, IL-20, and IL-24) by using soluble-IL-20R2-Fc fusion protein significantly ameliorated the severity of arthritis in CIA model [96]. These findings indicate multiple members of IL-20 subfamily may be involved in the pathogenesis of RA.

In 2017, a new oral JAK (Janus kinase) inhibitor –Tofacitinib (Xeljanz) was approved for the moderate and severe RA treatment [97]. In the treatment-refractory population, Tofacitinib significantly improved the disease severity and symptoms of RA patients [98]. The JAK receptor tyrosine kinases family binds to the cytokine receptors and elicits an inflammatory response inside cells [99, 100]. IL-20 is believed to activate JAK/STAT pathway to regulate its biological function. However, whether Tofacitinib efficiently inhibits IL-20 signaling needs to be further confirmed.

### Osteoporosis and bone fracture

Many skeletal diseases, such as osteoporosis and bone fracture, are results of the excess osteoclast activity or the decreased osteoblast activity. The current drugs for treating osteoporosis are designed to inhibit the osteoclastic bone resorption or to promote the osteoblastic bone formation, however, there is still no drug can reduce the severity of osteoporosis efficiently [86]. Currently, there aren’t any drugs available to boost bone formation to treat osteoporosis and bone fracture.

RANKL-RANK signaling is a determinant pathway for osteoclast differentiation. RANKL blockade, such as anti-RANKL mAb and OPG, inhibit the bone resorption by suppressing osteoclastogenesis [101, 102]. Denosumab (Amgen Inc.) is an anti-RANKL mAb to treat postmenopausal women with osteoporosis and bone metastasis [63, 103]. Sclerostin is a glycoprotein that inhibits the activation of osteoblasts. The sclerostin inhibitor – AMG785 (Amgen Inc.) has protective effects against osteoporosis in preclinical studies and is in the clinical trials for treating postmenopausal women with osteoporosis [63, 104, 105]. It should be noted that many factors have impacts on both bone formation and resorption. Anti-resorption agents alone treatment probably simultaneous inhibits bone formation, in turn, compromises the efficacy of the drug [106, 107]. Therefore, we need to search for novel therapeutic targets and develop new anti-osteoporotic drugs.

We previously showed that IL-20 regulates osteoclastogenesis through enhancing the RANK and cathepsin G expression and inducing the RANKL expression in osteoblasts. Therefore, IL-20 affects not only the formation of osteoclast but also influences the function of osteoblast. Furthermore, we found IL-20 also directly targets on osteoblasts to suppress osteoblastogenesis through inducing sclerostin expression and inhibiting RUNX2, OSX, Atf4, and OPG expression. In OVX-induced bone loss model, the anti-IL-20 mAb 7E suppresses osteoclasts differentiation and increases the BMD. Also, anti-IL-20 mAb 7E treatment promotes osteoblast differentiation and significantly increases the rate of bone healing in vivo. Therefore, the anti-IL-20 mAb 7E not only inhibits osteoclastogenesis but also promotes osteoblastogenesis. The result indicates that blocking of IL-20’s function is a potential way to treat osteoporosis and bone fracture, with more advantages than current drugs on the market.

### Cancer-induced osteolysis

Recently, breast cancer and prostate cancer have become the highest cancer incident around the world. These two types of cancer often cause bone metastasis. Dysregulations of osteoblastogenesis and osteoclastogenesis are a critical mechanism for cancer-induced osteolysis. We generally consider the bone microenvironment has an impact on the colonization of cancer cells and metastasis.

IL-20 induces the expression of MMP9, MMP-12, RANKL, cathepsin K and cathepsin G in cancer cells. The result suggested that IL-20 regulates many critical factors, which are involved in bone metastasis. In 4 T1 breast cancer-induced osteolytic bone metastasis model, anti-IL-20 mAb 7E treatment not only inhibited bone colonization but also prevented the osteolytic bone loss in mice. In PC-3 prostate cancer-induced osteolytic bone metastasis model, we found the similar effects, treatment of anti-IL-20 mAb 7E not only restrained prostate cancer growth but also protected against cancer-induced osteolysis. The data indicates anti-IL-20 mAb 7E is able to regulate the bone formation and form the microenvironment harmful to tumor cells. Thus, blocking IL-20 is a promising therapeutic strategy to halt breast tumor growth and bone colonization, in turn, prevent cancer-induced bone loss.

## Conclusions

**Fig. 1:**
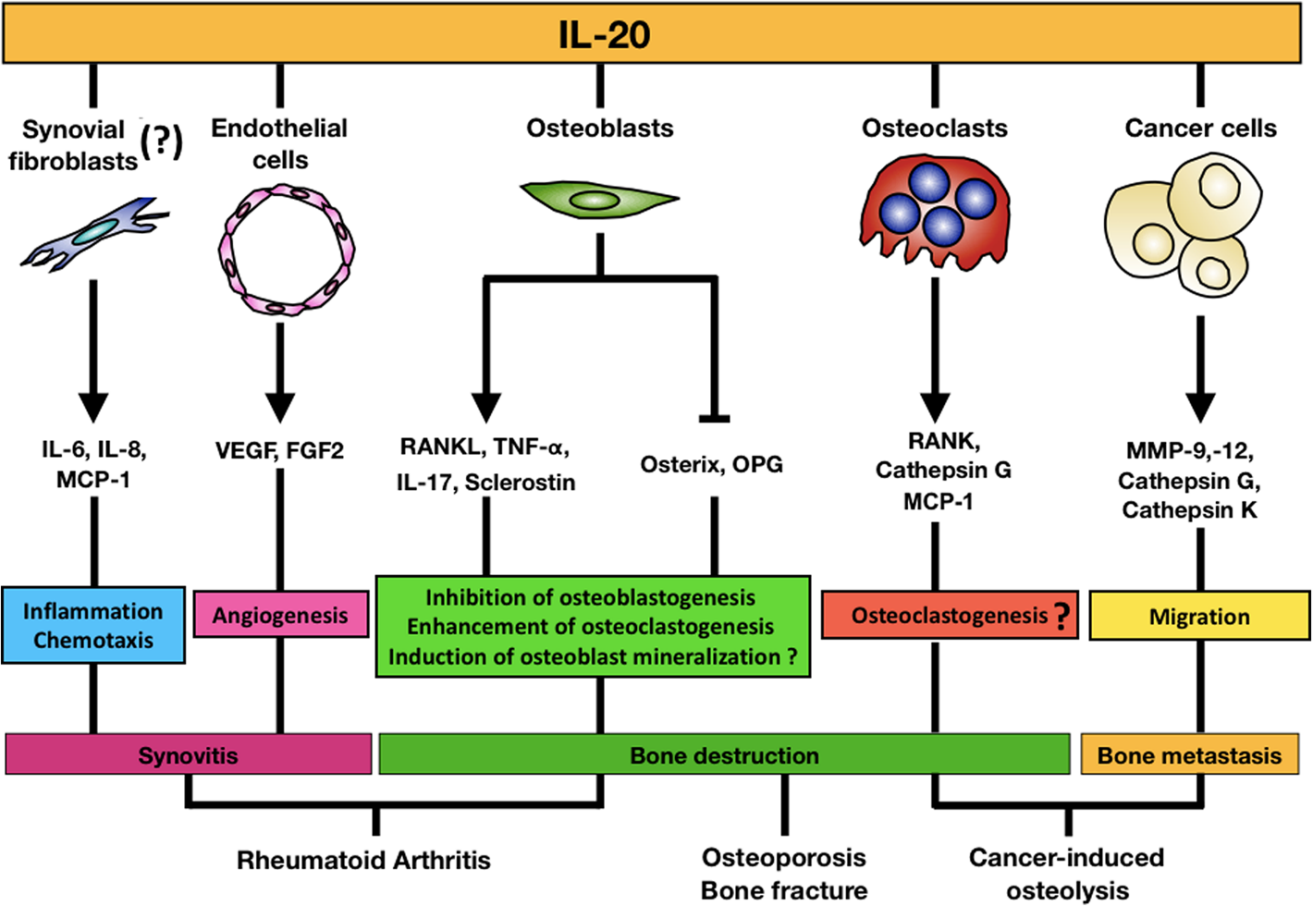
Working model of IL-20’s functions in bone-related diseases

We addressed the multifunctional roles of IL-20 involving in bone-related diseases in this review. There is a summarized working model that IL-20 implicated in the pathogenesis of these bone-related diseases (Fig. 1). IL-20 is a pleiotropic proinflammatory cytokine, which has the ability to enhance the inflammation, angiogenesis, and chemotaxis. In addition, IL-20 is a crucial mediator that regulating the balance between osteoclastogenesis and osteoblastogenesis. The observation makes us realize the novel biological functions of IL-20 in different cell types. Blocking IL-20’s function including monoclonal antibodies or small molecules may be a feasible treatment option for IL-20-mediated bone loss-related diseases in the future. Furthermore, the combined treatment of anti-IL-20 mAb with current drugs might be useful for these multifactorial diseases to slow down the progression and ameliorate their severity.

## Abbreviations

ALP: Alkaline phosphatase
BMD: Bone mineral density
CIA: Collagen-induced arthritis
CTX: C-terminal telopeptide of collagen
EMT: Epithelial-mesenchymal transition
ERK1/2: Extracellular signal-regulated kinase 1/2
FGF2: Fibroblast growth factor 2
HUVECs: Human umbilical vein endothelial cells
mAb: Monoclonal antibody
MCP-1: Monocyte chemoattractant protein-1
M-CSF: Macrophage colony-stimulating factor
MMPs: matrix metalloproteinases
OPG: osteoprotegerin
OSX: Osterix
OVX: Ovariectomy
RA: Rheumatoid arthritis
RANK: Receptor activator of nuclear factor kappa-B
RANKL: Receptor activator of nuclear factor kappa-B ligand
RASFs: Rheumatoid arthritis synovial fibroblasts
STAT3: Signal transducer and activator of transcription 3
TRAP: Tartrate-resistant acid phosphatase
VEGF: vascular endothelial growth factor

## References

[1] Vilcek J, Feldmann M (2004). Historical review: cytokines as therapeutics and targets of therapeutics. Trends Pharmacol Sci.

[2] Zhang JM, An J (2007). Cytokines, Inflammation, and pain. Int Anesthesiol Clin.

[3] Feldmann M (2008). Many cytokines are very useful therapeutic targets in disease. J Clin Invest.

[4] Turner MD, Nedjai B, Hurst T, Pennington DJ (2014). Cytokines and chemokines: at the crossroads of cell signalling and inflammatory disease. Biochim Biophys Acta.

[5] Dinarello CA (2000). Proinflammatory cytokines. Chest.

[6] Chan AC, Carter PJ (2010). Therapeutic antibodies for autoimmunity and inflammation. Nat Rev Immunol.

[7] Feng X, McDonald JM (2011). Disorders of bone remodeling. Annu Rev Pathol.

[8] Tanaka Y, Nakayamada S, Okada Y (2005). Osteoblasts and osteoclasts in bone remodeling and inflammation. Curr Drug Targets Inflamm Allergy.

[9] Rutkovskiy A, Stenslokken KO, Vaage IJ (2016). Osteoblast differentiation at a glance. Med Sci Monit Basic Res.

[10] Boyce BF, Xing L (2008). Functions of RANKL/RANK/OPG in bone modeling and remodeling. Arch Biochem Biophys.

[11] Mundy GR (1993). Cytokines and Growth factors in the regulation of bone remodeling. J Bone Miner Res.

[12] Wada T, Nakashima T, Hiroshi N, Penninger JM (2006). RANKL-RANK signaling in osteoclastogenesis and bone disease. Trends Mol Med.

[13] Goldring SR, Gravallese EM (2000). Mechanisms of bone loss in inflammatory arthritis: diagnosis and therapeutic implications. Arthritis Res.

[14] Manolagas SC, Jilka RL (1995). Bone marrow, cytokines, and bone remodeling. Emerging insights into the pathophysiology of osteoporosis. N Engl J Med.

[15] Blumberg H, Conklin D, Xu WF, Grossmann A, Brender T, Carollo S, Eagan M, Foster D, Haldeman BA, Hammond A, Haugen H, Jelinek L, Kelly JD, Madden K, Maurer MF, Parrish-Novak J, Prunkard D, Sexson S, Sprecher C, Waggie K, West J, Whitmore TE, Yao L, Kuechle MK, Dale BA, Chandrasekher YA (2001). Interleukin 20: discovery, receptor identification, and role in epidermal function. Cell.

[16] Stenderup K, Rosada C, Worsaae A, Dagnaes-Hansen F, Steiniche T, Hasselager E, Iversen LF, Zahn S, Woldike H, Holmberg HL, Romer J, Kragballe K, Clausen JT, Dam TN (2009). Interleukin-20 plays a critical role in maintenance and development of psoriasis in the human xenograft transplantation model. Br J Dermatol.

[17] Chen WY, Cheng BC, Jiang MJ, Hsieh MY, Chang MS (2006). IL-20 is expressed in atherosclerosis plaques and promotes atherosclerosis in apolipoprotein E-deficient mice. Arterioscler Thromb Vasc Biol.

[18] Chen WY, Chang MS (2009). IL-20 is regulated by hypoxia-inducible factor and up-regulated after experimental ischemic stroke. J Immunol.

[19] Hsieh MY, Chen WY, Jiang MJ, Cheng BC, Huang TY, Chang MS (2006). Interleukin-20 promotes angiogenesis in a direct and indirect manner. Genes Immun.

[20] Hanel KH, Cornelissen C, Luscher B, Baron JM (2013). Cytokines and the skin barrier. Int J Mol Sci.

[21] Hsu YH, Chiu YS, Chen WY, Huang KY, Jou IM, Wu PT, Wu CH, Chang MS. Anti-IL-20 monoclonal antibody promotes bone fracture healing through regulating IL-20-mediated osteoblastogenesis. Sci Rep. 2016;6(24339) 10.1038/srep24339 PMC4830982 27075747

[22] Hsu YH, Chen WY, Chan CH, Wu CH, Sun ZJ, Chang MS (2011). Anti-IL-20 monoclonal antibody inhibits the differentiation of osteoclasts and protects against osteoporotic bone loss. J Exp Med.

[23] Otkjaer K, Kragballe K, Johansen C, Funding AT, Just H, Jensen UB, Sorensen LG, Norby PL, Clausen JT, Iversen L (2007). IL-20 gene expression is induced by IL-1beta through mitogen-activated protein kinase and NF-kappaB-dependent mechanisms. J Invest Dermatol.

[24] Hsing CH, Ho CL, Chang LY, Lee YL, Chuang SS, Chang MS (2006). Tissue microarray analysis of interleukin-20 expression. Cytokine.

[25] Hsu YH, Li HH, Hsieh MY, Liu MF, Huang KY, Chin LS, Chen PC, Cheng HH, Chang MS (2006). Function of interleukin-20 as a proinflammatory molecule in rheumatoid and experimental arthritis. Arthritis Rheum.

[26] Hsu YH, Wei CC, Shieh DB, Chan CH, Chang MS (2012). Anti-IL-20 monoclonal antibody alleviates inflammation in oral cancer and suppresses tumor growth. Mol Cancer Res.

[27] Hsu YH, Hsing CH, Li CF, Chan CH, Chang MC, Yan JJ, Chang MS (2012). Anti-IL-20 monoclonal antibody suppresses breast cancer progression and bone osteolysis in murine models. J Immunol.

[28] Wegenka UM (2010). IL-20: biological functions mediated through two types of receptor complexes. Cytokine Growth Factor Rev.

[29] Stenderup K, Rosada C, Worsaae A, Clausen JT, Norman Dam T (2007). Interleukin-20 as a target in psoriasis treatment. Ann N Y Acad Sci.

[30] Rich BE, Kupper TS (2001). Cytokines: IL-20 - a new effector in skin inflammation. Curr Biol.

[31] Li HH, Hsu YH, Wei CC, Lee PT, Chen WC, Chang MS (2008). Interleukin-20 induced cell death in renal epithelial cells and was associated with acute renal failure. Genes Immun.

[32] Chiu YS, Wei CC, Lin YJ, Hsu YH, Chang MS (2014). IL-20 and IL-20R1 antibodies protect against liver fibrosis. Hepatology.

[33] Huang KY, Lin RM, Chen WY, Lee CL, Yan JJ, Chang MS (2008). IL-20 may contribute to the pathogenesis of human intervertebral disc herniation. Spine.

[34] Deshmane SL, Kremlev S, Amini S, Sawaya BE (2009). Monocyte chemoattractant protein-1 (MCP-1): an overview. J Interf Cytokine Res.

[35] Wei CC, Hsu YH, Li HH, Wang YC, Hsieh MY, Chen WY, Hsing CH, Chang MS (2006). IL-20: biological functions and clinical implications. J Biomed Sci.

[36] Li A, Dubey S, Varney ML, Dave BJ, Singh RK (2003). IL-8 directly enhanced endothelial cell survival, proliferation, and matrix metalloproteinases production and regulated angiogenesis. J Immunol.

[37] Lee SJ, Cho SC, Lee EJ, Kim S, Lee SB, Lim JH, Choi YH, Kim WJ, Moon SK (2013). Interleukin-20 promotes migration of bladder cancer cells through extracellular signal-regulated kinase (ERK)-mediated MMP-9 protein expression leading to nuclear factor (NF-kappaB) activation by inducing the up-regulation of p21(WAF1) protein expression. J Biol Chem.

[38] Gravallese EM (2002). Bone destruction in arthritis. Ann Rheum Dis.

[39] Odeh M (1997). New insights into the pathogenesis and treatment of rheumatoid arthritis. Clin Immunol Immunopathol.

[40] Huber LC, Distler O, Tarner I, Gay RE, Gay S, Pap T (2006). Synovial fibroblasts: key players in rheumatoid arthritis. Rheumatology (Oxford).

[41] Szekanecz Z, Besenyei T, Paragh G, Koch AE (2009). Angiogenesis in rheumatoid arthritis. Autoimmunity.

[42] Bodolay E, Koch AE, Kim J, Szegedi G, Szekanecz Z (2002). Angiogenesis and chemokines in rheumatoid arthritis and other systemic inflammatory rheumatic diseases. J Cell Mol Med.

[43] Senolt L, Prajzlerova K, Hulejova H, Sumova B, Filkova M, Veigl D, Pavelka K, Vencovsky J (2017). Interleukin-20 is triggered by TLR ligands and associates with disease activity in patients with rheumatoid arthritis. Cytokine.

[44] Valentina M, Jan F, Peder NL, Bo Z, Hongjie D, Pernille K. Cytokine detection and simultaneous assessment of rheumatoid factor interference in human serum and synovial fluid using high-sensitivity protein arrays on plasmonic gold chips. BMC Biotechnol. 2015;15(73) 10.1186/s12896-015-0186-0 PMC4535377 26268325

[45] Kragstrup TW, Otkjaer K, Holm C, Jorgensen A, Hokland M, Iversen L, Deleuran B (2008). The expression of IL-20 and IL-24 and their shared receptors are increased in rheumatoid arthritis and spondyloarthropathy. Cytokine.

[46] Smeets T.J.M., Chandrasekher Y., Haringman J.J. and Tak P.P. IL-20 is expressed in inflamed synovium of patients with psoriatic arthritis and rheumatoid arthritis. Arthritis Research & Therapy 6:S9-S9, 2004.

[47] Geusens P (2012). The role of RANK ligand/osteoprotegerin in rheumatoid arthritis. Ther Adv Musculoskelet Dis.

[48] Burrage PS, Mix KS, Brinckerhoff CE (2006). Matrix metalloproteinases: role in arthritis. Front Biosci.

[49] Hsu YH, Chang MS (2010). Interleukin-20 antibody is a potential therapeutic agent for experimental arthritis. Arthritis Rheum.

[50] Gaffen SL (2009). The role of interleukin-17 in the pathogenesis of rheumatoid arthritis. Curr Rheumatol Rep.

[51] Hsu YH, Chang MS (2017). IL-20 in rheumatoid arthritis. Drug Discov Today.

[52] Ross FP, Teitelbaum SL (2005). alphavbeta3 and macrophage colony-stimulating factor: partners in osteoclast biology. Immunol Rev.

[53] Takayanagi H, Sato K, Takaoka A, Taniguchi T (2005). Interplay between interferon and other cytokine systems in bone metabolism. Immunol Rev.

[54] Kragstrup TW, Greisen SR, Nielsen MA, Rhodes C, Stengaard-Pedersen K, Hetland ML, Horslev-Petersen K, Junker P, Ostergaard M, Hvid M, Vorup-Jensen T, Robinson WH, Sokolove J, Deleuran B. The interleukin-20 receptor axis in early rheumatoid arthritis: novel links between disease-associated autoantibodies and radiographic progression. Arthritis Res Ther. 2016;18(61) 10.1186/s13075-016-0964-7 PMC4788924 26968800

[55] Wilson TJ, Nannuru KC, Singh RK (2009). Cathepsin G recruits osteoclast precursors via proteolytic activation of protease-activated receptor-1. Cancer Res.

[56] Tao Z, Shi A, Lu C, Song T, Zhang Z, Zhao J (2015). Breast Cancer: epidemiology and etiology. Cell Biochem Biophys.

[57] Redig AJ, McAllister SS (2013). Breast cancer as a systemic disease: a view of metastasis. J Intern Med.

[58] Mansour M, Teo ZL, Luen SJ, Loi S (2017). Advancing immunotherapy in metastatic breast Cancer. Curr Treat Options in Oncol.

[59] Patel LR, Camacho DF, Shiozawa Y, Pienta KJ, Taichman RS (2011). Mechanisms of cancer cell metastasis to the bone: a multistep process. Future Oncol.

[60] Dong Z, Bonfil RD, Chinni S, Deng X, Trindade Filho JC, Bernardo M, Vaishampayan U, Che M, Sloane BF, Sheng S, Fridman R, Cher ML (2005). Matrix metalloproteinase activity and osteoclasts in experimental prostate cancer bone metastasis tissue. Am J Pathol.

[61] Kessenbrock K, Plaks V, Werb Z (2010). Matrix metalloproteinases: regulators of the tumor microenvironment. Cell.

[62] Leng RX, Pan HF, Tao JH, Ye DQ (2011). IL-19, IL-20 and IL-24: potential therapeutic targets for autoimmune diseases. Expert Opin Ther Targets.

[63] Lewiecki EM (2011). New targets for intervention in the treatment of postmenopausal osteoporosis. Nat Rev Rheumatol.

[64] Litwin MS, Tan HJ (2017). The diagnosis and treatment of prostate Cancer: a review. JAMA.

[65] Manca P, Pantano F, Iuliani M, Ribelli G, De Lisi D, Danesi R, Del Re M, Vincenzi B, Tonini G, Santini D (2017). Determinants of bone specific metastasis in prostate cancer. Crit Rev Oncol Hematol.

[66] Heerboth S, Housman G, Leary M, Longacre M, Byler S, Lapinska K, Willbanks A, Sarkar S. EMT and tumor metastasis. Clin Transl Med. 2015;4(6) 10.1186/s40169-015-0048-3 PMC4385028 25852822

[67] Micalizzi DS, Farabaugh SM, Ford HL (2010). Epithelial-mesenchymal transition in cancer: parallels between normal development and tumor progression. J Mammary Gland Biol Neoplasia.

[68] Jing Y, Han Z, Zhang S, Liu Y, Wei L (2011). Epithelial-mesenchymal transition in tumor microenvironment. Cell Biosci.

[69] Tsai JH, Yang J (2013). Epithelial-mesenchymal plasticity in carcinoma metastasis. Genes Dev.

[70] Roodman GD (2001). Biology of osteoclast activation in cancer. J Clin Oncol.

[71] Le Pape F, Vargas G, Clezardin P (2016). The role of osteoclasts in breast cancer bone metastasis. J Bone Oncol.

[72] Goto T, Yamaza T, Tanaka T (2003). Cathepsins in the osteoclast. J Electron Microsc.

[73] Le Gall C, Bonnelye E, Clezardin P (2008). Cathepsin K inhibitors as treatment of bone metastasis. Curr Opin Support Palliat Care.

[74] Wilson TJ, Nannuru KC, Futakuchi M, Sadanandam A, Singh RK (2008). Cathepsin G enhances mammary tumor-induced osteolysis by generating soluble receptor activator of nuclear factor-kappaB ligand. Cancer Res.

[75] Tan GJ, Peng ZK, Lu JP, Tang FQ (2013). Cathepsins mediate tumor metastasis. World J Biol Chem.

[76] Hsu YH, Wu CY, Hsing CH, Lai WT, Wu LW, Chang MS (2015). Anti-IL-20 monoclonal antibody suppresses prostate Cancer growth and bone Osteolysis in murine models. PLoS One.

[77] Einhorn TA, Gerstenfeld LC (2015). Fracture healing: mechanisms and interventions. Nat Rev Rheumatol.

[78] Marsell R, Einhorn TA (2011). The biology of fracture healing. Injury.

[79] Raggatt LJ, Partridge NC (2010). Cellular and molecular mechanisms of bone remodeling. J Biol Chem.

[80] Kragstrup TW, Andersen MN, Schiottz-Christensen B, Jurik AG, Hvid M, Deleuran B (2017). Increased interleukin (IL)-20 and IL-24 target osteoblasts and synovial monocytes in spondyloarthritis. Clin Exp Immunol.

[81] Lewiecki EM (2014). Role of sclerostin in bone and cartilage and its potential as a therapeutic target in bone diseases. Ther Adv Musculoskelet Dis.

[82] MacNabb C, Patton D, Hayes JS (2016). Sclerostin antibody therapy for the treatment of osteoporosis: clinical prospects and challenges. J Osteoporos.

[83] Siebert S, Tsoukas A, Robertson J, McInnes I (2015). Cytokines as therapeutic targets in rheumatoid arthritis and other inflammatory diseases. Pharmacol Rev.

[84] Maini RN, Elliott MJ, Brennan FM, Williams RO, Chu CQ, Paleolog E, Charles PJ, Taylor PC, Feldmann M (1995). Monoclonal anti-TNF alpha antibody as a probe of pathogenesis and therapy of rheumatoid disease. Immunol Rev.

[85] van den Berg WB (2001). Anti-cytokine therapy in chronic destructive arthritis. Arthritis Res.

[86] Hsu YH, Chang MS (2014). The therapeutic potential of anti-interleukin-20 monoclonal antibody. Cell Transplant.

[87] Andersson C, Serikawa K, Pelzer H, Thygesen P, Smith P, Kruse K, Biswas S, Fox B, Milner A, Kvist P, Hebsgaard J, Pass J, Romer J. IL-20 is not involved in mouse collagen induced arthritis. Arthritis Rheum-Us. 2012;64(10):S880–0.

[88] Song YW, Kang EH (2010). Autoantibodies in rheumatoid arthritis: rheumatoid factors and anticitrullinated protein antibodies. QJM.

[89] Othman MA, Ghazali WS, Yahya NK, Wong KK (2016). Correlation of demographic and clinical characteristics with rheumatoid factor Seropositivity in rheumatoid arthritis patients. Malays J Med Sci.

[90] Braschi E, Shojania K, Allan GM (2016). Anti-CCP: a truly helpful rheumatoid arthritis test?. Can Fam Physician.

[91] Senolt L, Leszczynski P, Dokoupilova E, Gothberg M, Valencia X, Hansen BB, Canete JD (2015). Efficacy and safety of anti-Interleukin-20 monoclonal antibody in patients with rheumatoid arthritis: a randomized phase IIa trial. Arthritis Rheumatol.

[92] Chalan P, Bijzet J, van den Berg A, Kluiver J, Kroesen BJ, Boots AM, Brouwer E (2016). Analysis of serum immune markers in seropositive and seronegative rheumatoid arthritis and in high-risk seropositive arthralgia patients. Sci Rep.

[93] Rutz S, Wang X, Ouyang W (2014). The IL-20 subfamily of cytokines--from host defence to tissue homeostasis. Nat Rev Immunol.

[94] Hsu YH, Hsieh PP, Chang MS (2012). Interleukin-19 blockade attenuates collagen-induced arthritis in rats. Rheumatology (Oxford).

[95] Kragstrup TW (2016). The IL-20 receptor axis in immune-mediated inflammatory arthritis: novel links between innate immune recognition and bone homeostasis. Scand J Rheumatol.

[96] Liu X, Zhou H, Huang X, Cui J, Long T, Xu Y, Liu H, Yu R, Zhao R, Luo G, Huang A, Liang JG, Liang P (2016). A broad blockade of signaling from the IL-20 family of cytokines potently attenuates collagen-induced arthritis. J Immunol.

[97] Dhillon S (2017). Tofacitinib: a review in rheumatoid arthritis. Drugs.

[98] Burmester GR, Blanco R, Charles-Schoeman C, Wollenhaupt J, Zerbini C, Benda B, Gruben D, Wallenstein G, Krishnaswami S, Zwillich SH, Koncz T, Soma K, Bradley J, Mebus C, investigators OS (2013). Tofacitinib (CP-690,550) in combination with methotrexate in patients with active rheumatoid arthritis with an inadequate response to tumour necrosis factor inhibitors: a randomised phase 3 trial. Lancet.

[99] Tanaka Y, Maeshima K, Yamaoka K (2012). In vitro and in vivo analysis of a JAK inhibitor in rheumatoid arthritis. Ann Rheum Dis.

[100] Hsu L, Armstrong AW (2014). JAK inhibitors: treatment efficacy and safety profile in patients with psoriasis. J Immunol Res.

[101] Jules J, Ashley JW, Feng X (2010). Selective targeting of RANK signaling pathways as new therapeutic strategies for osteoporosis. Expert Opin Ther Targets.

[102] McClung M (2007). Role of RANKL inhibition in osteoporosis. Arthritis Res Ther.

[103] Lewiecki EM (2009). Denosumab for the treatment of postmenopausal osteoporosis. Womens Health (Lond).

[104] McClung MR (2017). Sclerostin antibodies in osteoporosis: latest evidence and therapeutic potential. Ther Adv Musculoskelet Dis.

[105] Lewiecki EM (2011). Sclerostin monoclonal antibody therapy with AMG 785: a potential treatment for osteoporosis. Expert Opin Biol Ther.

[106] Odvina CV, Zerwekh JE, Rao DS, Maalouf N, Gottschalk FA, Pak CY (2005). Severely suppressed bone turnover: a potential complication of alendronate therapy. J Clin Endocrinol Metab.

[107] Rachner TD, Khosla S, Hofbauer LC (2011). Osteoporosis: now and the future. Lancet.

